# A Comprehensive Review of Pediatric Obstructive Sleep Apnea: From Assessment to Intervention

**DOI:** 10.7759/cureus.78051

**Published:** 2025-01-27

**Authors:** Ulhas Jadhav, Jay Bhanushali, Arman Sindhu, Bingu Shiv Kiran Reddy, Amit Toshniwal, Mummaneni Rashmika

**Affiliations:** 1 Respiratory Medicine, Jawaharlal Nehru Medical College, Datta Meghe Institute of Higher Education and Research, Wardha, IND; 2 Pulmonary and Critical Care Medicine, Jawaharlal Nehru Medical College, Datta Meghe Institute of Higher Education and Research, Wardha, IND

**Keywords:** childhood behaviour, childhood obesity, hypertrophic adenoids, sleep apnea and hypertension, sleep disorder breathing, snoring

## Abstract

Pediatric obstructive sleep apnea (OSA) is a prevalent sleep disorder characterized by upper airway obstruction during sleep, leading to oxygen desaturation and sleep disruptions. This comprehensive review examines the epidemiology, pathophysiology, clinical manifestations, diagnostic approaches, consequences, management strategies, challenges, and future directions of pediatric OSA. Prevalence rates vary globally, with notable associations found between OSA and obesity. The review explores the anatomical and neuromuscular factors contributing to airway obstruction, emphasizing the importance of timely identification and intervention to mitigate long-term health implications. Diagnostic tools such as polysomnography (PSG) are essential, although challenges in accessibility exist. Pediatric OSA is linked to various health issues, including cardiovascular complications, metabolic disturbances, and neurocognitive impairments. Treatment options range from surgical interventions like adenotonsillectomy to non-pharmacological therapies and pharmacotherapy. Challenges in diagnosis and treatment warrant research into innovative diagnostic approaches and refining clinical prediction rules. Addressing these areas will enhance clinical management and outcomes for children with OSA.

## Introduction and background

Pediatric obstructive sleep apnea (OSA) presents as a disruption in the upper airway function during sleep, leading to either partial or complete blockage and consequent drops in oxygen saturation or sleep interruptions. This condition affects more than just sleep; it impacts childhood behavior, neurodevelopment, metabolic processes, and overall well-being. Early detection, comprehensive evaluation, and effective treatment are essential to prevent potential long-term health and developmental consequences [1]. Estimates suggest that pediatric OSA affects about one to three percent of children, posing significant clinical challenges due to its impact on individual health. If left untreated, pediatric OSA can result in metabolic, endocrine, cardiovascular, and neurobehavioral issues, potentially leading to lasting health problems [2].

The global prevalence of pediatric OSA varies. Recent research shows that obesity increases the risk by four to five times. Among healthy children, the prevalence of OSA is estimated to be between one to three percent. In Japan, the prevalence among children aged six to eight years is about four percent, according to the International Criteria of Sleep Disorders version II (ICSD II) diagnostic criteria [3]. In Italy, severe OSA affects two to three percent of children, with the prevalence of all obstructive sleep-disordered breathing (OSDB) ranging from eight to eleven percent, figures that are consistent with international findings [4-6]. In India, the prevalence of pediatric OSA varies across different studies. Reports suggest a prevalence of about nine percent among school children [7], fourteen percent among adolescent orthodontic patients [8], and nine percent in community-based studies [9]. Additionally, studies in urban Indian males indicate a prevalence of 19.5 percent for sleep-disordered breathing (SDB) and 7.5 percent for obstructive sleep apnea-hypopnea syndrome (OSAHS) [10]. 

## Review

Understanding the pathophysiology of pediatric obstructive sleep apnea

The pediatric upper airway anatomy undergoes significant changes as children grow. Initially, features such as a relatively prominent occiput, a high laryngeal position, and a U-shaped epiglottis make the pediatric airway prone to obstruction. Over time, the dimensions of the upper airway and the surrounding soft tissues increase, improving airflow. By around age eight, the pediatric airway reaches a level of maturation that closely resembles the adult airway in both structure and function [11,12]. During sleep, upper airway obstruction often occurs due to negative collapsing pressure during inspiration, although progressive expiratory narrowing in the retro palatal area also plays a significant role. The extent of upper airway narrowing during sleep often correlates with body mass index, suggesting that both anatomical and neuromuscular factors contribute to airway obstruction [13].

During sleep, the upper airway may collapse due to various factors, including anatomical, mechanical, and neuro-functional conditions. Factors such as the efficiency of upper airway dilating muscles, ventilatory motor output, and changes in respiratory muscle activity induced by sleep can affect upper airway patency [14]. Increased upper airway resistance during sleep due to decreased muscle activity can lead to restricted inspiratory flow and eventual complete occlusion of the upper airway. Sleep-related breathing disorders, such as obstructive sleep apnea, result from recurrent episodes of pharyngeal airway obstruction, leading to repeated episodes of oxygen deprivation and arousal from sleep [14,15].

Adenotonsillar hypertrophy, craniofacial anomalies, and obesity are significant risk factors for the development of obstructive sleep apnea (OSA). In children, adenotonsillar hypertrophy is the predominant cause of OSA, whereas in adults, factors like obesity, male gender, and aging are crucial for OSA onset [16]. Craniofacial abnormalities such as micrognathia and midface hypoplasia further increase the risk of OSA. These factors collectively cause upper airway constriction, leading to pharyngeal collapse during sleep and contributing to the development of OSA [16,17].

Clinical manifestations and diagnostic approach

Sleep apnea manifests in two primary forms: central and obstructive. Central sleep apnea arises from dysfunction in the central nervous system, resulting in a lack of respiratory drive without corresponding respiratory effort. Conversely, obstructive sleep apnea (OSA), which accounts for 95% of cases, is caused by complete or partial upper airway collapse during sleep, leading to frequent arousals or significant oxygen desaturation [18]. Factors contributing to upper airway narrowing or instability include anatomical, genetic, or neuromuscular issues. These may involve intrinsic elements like critical airway pressure and extrinsic factors such as adipose tissue deposition, tissue hypertrophy, and craniofacial anomalies, all of which increase the risk of airway collapse [18,19].

Parents often report symptoms such as snoring, mouth breathing, observed apneic episodes, frequent nighttime awakenings, and secondary nocturnal enuresis in children with OSA. These symptoms disrupt the child's sleep, leading to behavioral issues like hyperactivity, irritability, or aggression, prompting medical evaluation [18]. Clinical examination may reveal signs such as fatigue, hyperactivity, "allergic shiners," swollen nasal mucosa, micrognathia, macroglossia, a high-arched palate, adenoidal facies, or enlarged tonsils [18].

Hyponasal speech and nasal congestion may also be evident. Childhood obesity is a growing risk factor for pediatric OSA, highlighting the importance of routine screening for height, weight, and body mass index (BMI) during pediatric visits. Notably, each unit increase in BMI above the 50th percentile corresponds to a 12% increase in the risk of developing OSA [18].

Nocturnal polysomnography (PSG) is the gold standard for diagnosing OSA, but its use can be limited by cost, time, and resource availability. Home overnight oximetry can provide supplementary information but cannot replace PSG for diagnosis [19]. For suspected cardiopulmonary issues, additional evaluations like chest X-rays and electrocardiograms (EKGs) are necessary, with cardiac assessments extending to echocardiograms before considering surgery for severe OSA in children [18]. Measuring specific inflammatory biomarkers, such as kallikrein-1, uromodulin, urocortin-3, and orosomucoid-1, can offer insights, as they may be elevated in children with OSA. Routine laboratory tests, including complete blood count (CBC), iron studies, and thyroid-stimulating hormone (TSH) levels, are also recommended to rule out other causes of sleep disturbances [18]. Imaging studies can help evaluate anatomical abnormalities contributing to OSA and are most effective when used alongside PSG for a comprehensive diagnosis [18-20].

During PSG, various parameters are assessed to evaluate sleep quality and quantity, including monitoring brain activity, heart rate, airflow through the nose and mouth, blood oxygen levels, limb movements, eye movements, and snoring patterns using specialized sensors. These data enable the calculation of several sleep metrics, such as sleep onset latency, sleep efficiency, and duration in each sleep stage [1]. A crucial metric in diagnosing OSA is the apnea/hypopnea index (AHI), which represents the average number of apnea and hypopnea episodes per hour of sleep. AHI scores of 1 to 4.9 events per hour indicate mild OSA, 5 to 9.9 events per hour suggest moderate OSA and more than nine events per hour signify severe OSA. In children up to 13 years old, an AHI of 1 or higher is considered abnormal, although the clinical significance of AHI values between 1 and 1.9 events per hour remains debated [1,18].

OSA in children is associated with various health issues, including behavioral and neurocognitive problems, growth impairments, cardiovascular complications, and metabolic disturbances. In-laboratory PSG remains the gold standard for diagnosing pediatric OSA, but access to sleep centers, specialized training requirements, and logistical challenges can hinder timely diagnosis and treatment. Alternative diagnostic methods, such as home sleep testing and wearable technology, are gaining attention as potential solutions to these limitations [21]. Pediatric OSA can mimic symptoms of allergic rhinitis, ADHD, developmental delay, gastroesophageal reflux, primary nocturnal enuresis, morning headaches, parasomnias, and narcolepsy. Polysomnography is essential for accurate diagnosis, differentiating OSA from these conditions [19].

Consequences and associated conditions of pediatric obstructive sleep apnea

Untreated pediatric OSA poses significant risks, including the potential development of pulmonary hypertension and right heart failure due to sustained hypoxia. Cognitive dysfunction, learning impairments, and academic underperformance are expected consequences of undiagnosed or untreated pediatric OSA. Younger children may experience failure to thrive due to the increased work of breathing associated with the condition [18]. Pediatric OSA is intricately connected with metabolic and endocrine ramifications. Beyond its correlation with obesity, OSA can independently trigger insulin resistance and metabolic disturbances. It also influences branched-chain amino acid metabolism. OSA in children alters metabolic parameters such as insulin resistance, fasting glucose, and lipid levels, posing significant risks for broader health complications [22,23].

Pediatric OSA carries significant cardiovascular implications. Children affected by OSA often exhibit elevated pulmonary arterial pressure, ventricular hypertrophy, and systemic hypertension, particularly among those with a high apnea-hypopnea index. They face an increased risk of major adverse cardiovascular events compared to peers without OSA. Despite these risks, the prevalence of pulmonary hypertension in pediatric OSA patients remains relatively low [24-26]. Pediatric OSA poses enduring consequences and comorbidities, influencing both the well-being and academic performance of affected children. It exerts a detrimental effect on the quality of life for both children and their families, with notable improvements observed post-adenotonsillectomy [27]. The severity of OSA correlates with increased familial distress, particularly concerning financial strain. Additionally, OSA contributes to behavioral and emotional challenges in children, which demonstrate improvement following appropriate treatment [27].

Approaches to managing pediatric obstructive sleep apnea

Non-pharmacological and Surgical Interventions

Adenotonsillectomy (A&T) is the primary treatment for children with enlarged adenoids or tonsils, while alternative surgical options like partial tonsillectomy and lingual tonsillectomy are considered in specific cases. In certain populations, craniofacial or bariatric surgery may be warranted, while tracheostomy remains a last resort [28,29]. Positive airway pressure (PAP) emerges as the most effective non-surgical therapy, suitable even for severe cases of OSA. Select populations with dental issues may benefit from rapid maxillary expansion or dental appliances. Therapies like positional therapy, supplemental oxygen, and weight loss have limited efficacy in most pediatric cases [28]. More emphasis on dietary restrictions and multi-disciplinary action with the help of a pediatrician, nutritionist, and bariatric physician must be taken against childhood obesity-related pediatric OSA. 

As per the guidelines from the American Academy of Otolaryngology-Head and Neck Surgery Foundation (AAO-HNSF), children aged two to 18 years with sleep-disordered breathing (SDB) should undergo polysomnography (PSG) before a tonsillectomy [30]. The most frequent reasons for PSG evaluation before the procedure include conditions such as Down syndrome, craniofacial abnormalities, obesity, neuromuscular disorders, mucopolysaccharidosis, sickle cell disease, symptoms that do not align with physical findings, and an unclear medical history [31]. Partial tonsillectomy is another surgical option with reduced postoperative complications and shorter recovery periods. However, studies indicate tonsillar regrowth rates ranging from 7.2% to 16.6%, raising concerns about long-term efficacy. Lingual tonsillectomy and uvulopalatopharyngoplasty, although utilized in some cases, lack substantial data supporting their superiority over other surgical methods [28]. Before A&T for severe pediatric OSA, PAP therapy should be evaluated during the perioperative phase, especially if the child is not suitable for surgery or continues to exhibit moderate to severe OSA post-surgery. Ensuring compliance with PAP therapy can be challenging for children, and prolonged use of the same mask may potentially alter facial structure over time [18].

Myofunctional therapy represents a novel approach to the management of OSA across both pediatric and adult populations. It involves retraining the oral cavity and oropharyngeal muscles, along with optimizing tongue positioning. While research on its effectiveness in pediatric OSA remains limited, with only small-scale studies conducted thus far, it holds promise as a potential adjunctive therapy in the treatment of OSA [32].

Pharmacotherapy

In managing pediatric OSA, various treatment modalities are employed, including intranasal steroids, montelukast, and nasal positive airway pressure (nPAP). Intranasal steroids such as fluticasone and budesonide have demonstrated efficacy in improving the apnea-hypopnea index (AHI) in children with OSA. Montelukast, classified as a leukotriene receptor antagonist, has shown notable improvements in polysomnography parameters and symptom reduction in pediatric OSA patients. The combined use of intranasal steroids and leukotriene inhibitors has exhibited enhanced efficacy compared to monotherapy, highlighting the potential synergistic benefits of combination therapy [33-35].

Combination Therapies

A combined approach to treating pediatric OSA involves the use of intranasal corticosteroids alongside oral montelukast, showcasing promising outcomes in over 80% of affected children, with notable improvements seen in sleep parameters normalization in 62% of cases. This combination therapy demonstrates superior efficacy compared to individual drug administration, emphasizing the potential synergistic benefits of combining intranasal corticosteroids and oral montelukast in managing pediatric OSA [35].

Challenges and future directions

The current diagnostic landscape for pediatric OSA faces challenges due to limited access to pediatric sleep laboratories and the demanding nature of diagnostic procedures. There is a pressing need for more explicit diagnostic criteria to effectively identify instances of partial airway obstruction in pediatric OSA [36]. Treatment approaches for pediatric OSA exhibit variability, with some children undergoing adenotonsillectomy solely based on clinical presentation, often without formal sleep assessment. Despite adenotonsillectomy, approximately 20% of children continue to experience persistent OSA. Furthermore, the reliability of clinical symptoms and signs alone in predicting pediatric OSA remains modest, underscoring the necessity for alternative diagnostic paradigms to enhance diagnostic precision [37,38].

Research in pediatric OSA could explore novel diagnostic avenues, such as transcriptomics and proteomics, to enhance diagnostic precision and uncover new biomarkers. Prioritizing research topics, including assessing the cost-effectiveness of management strategies and validating clinical prediction rules, could offer valuable insights for future investigations in pediatric OSA [38]. Examining the intricate interplay between critical illness, child development, and family dynamics, alongside longitudinal outcomes research, emerges as a pivotal area for advancing pediatric critical care research [38]. Further studies should be done on the implications of pediatric OSA on adult life.

## Conclusions

In conclusion, this review highlights the complexity of pediatric obstructive sleep apnea (OSA) and its extensive impact on children's health. Although adenotonsillectomy is the primary treatment, other modalities, such as positive airway pressure therapy and pharmacological interventions, also show potential. Challenges remain in accurately diagnosing and managing pediatric OSA. Future research should prioritize innovative diagnostic methods, refine clinical prediction rules, and investigate long-term outcomes and the cost-effectiveness of various management strategies. Addressing these areas will enhance clinical practice and improve outcomes for children with OSA. 

When obstructive sleep apnea (OSA) is suspected in a child, a comprehensive evaluation combining clinical assessment and diagnostic testing is essential. Key components of this evaluation include a multidisciplinary team approach, children with suspected OSA should be managed by a team comprising a pediatrician, an otolaryngologist (ear, nose, and throat (ENT) specialist), an orthodontist, and a speech therapist. Overnight polysomnography (PSG) is the gold standard for diagnosing OSA in children. Data obtained from diagnostic evaluations should be meticulously analyzed, considering the child's age, medical history, and specific symptoms, to ensure accurate diagnosis and appropriate management. Early detection and intervention are crucial to mitigate potential complications associated with pediatric OSA, such as behavioral issues, learning difficulties, and cardiovascular problems.
